# Characterization and Comparison of The “Frequent Exacerbator Phenotype” in Non-Cystic Fibrosis Bronchiectasis Patients in a TB-Endemic Country: A Hospital-Based Comparative Study

**DOI:** 10.4314/ejhs.v35i1.7S

**Published:** 2025-12

**Authors:** Shalom Kassahun Bekele, Weys Nesru Neda, Thomas Asfaw Atnafu, Hable Dessalegn Yigzaw, Hiwot Yegnaneh Anley, Eden Haile Hagos, Hanan Yusuf

**Affiliations:** 1 Department of Internal Medicine, School of Medicine, Addis Ababa University, Addis Ababa, Ethiopia; 2 Department of Women and Child Health, Uppsala University, Uppsala, Sweden

**Keywords:** Non–Cystic Fibrosis Bronchiectasis, Frequent Exacerbators, Phenotype, Risk Factors, Ethiopia

## Abstract

**Background:**

Bronchiectasis is a chronic lung disease characterized by permanent dilatation of the bronchi. Frequent exacerbations are associated with accelerated lung function decline and increased mortality risk. This study aimed to identify factors associated with the frequent exacerbator phenotype among patients with non–cystic fibrosis (non-CF) bronchiectasis receiving follow-up care in Ethiopia.

**Methods:**

A comparative institution-based study was conducted among 114 patients with non-CF bronchiectasis attending follow-up clinics. Based on the number of exacerbations in the preceding year, participants were categorized into two groups: frequent exacerbators (≥3 exacerbations per year; n = 34) and non-frequent exacerbators (<3 exacerbations per year; n = 80). Data were collected through structured interviews and review of electronic medical records.

**Results:**

The mean age of participants was 49.5 years (SD ±15.1). Most participants were female (55%) and residents of Addis Ababa (66%). Frequent exacerbators constituted 30% of the study population. Factors independently associated with frequent exacerbations included age >65 years (AOR = 3.7, 95% CI: 2.89–19.47), residence outside Addis Ababa (AOR = 9.7, 95% CI: 2.35–40.07), lack of formal education (AOR = 17.2, 95% CI: 1.78–66.98), smoking (AOR = 6.2, 95% CI: 1.25–31.15), resting oxygen saturation <88% (AOR = 10.1, 95% CI: 1.87-54.73), comorbid asthma (AOR = 4.3, 95% CI: 1.34-16.41), and higher dyspnea grades (grade III: AOR = 8.6; grade IV: AOR = 14.2).

**Conclusion:**

Approximately one-third of patients with bronchiectasis attending follow-up at Tikur Anbessa Specialized Hospital experienced frequent exacerbations. Advanced age, residence outside Addis Ababa, low educational status, smoking, hypoxemia, comorbid asthma, and severe dyspnea were significant determinants of the frequent exacerbator phenotype.

## Introduction

Bronchiectasis is a chronic lung disease associated with permanent dilatation of the bronchi and usually excess mucopurulent secretions (1). The term non-CF bronchiectasis has been used to describe a group of patients with bronchiectasis caused by conditions other than cystic fibrosis (CF), including post-infectious etiologies (e.g. following pneumonia, pertussis, or Mycobacterium infection), connective tissue diseases, allergic bronchopulmonary aspergillosis, immunodeficiency, and autoimmune conditions (2,5). The incidence and prevalence of bronchiectasis have increased in recent years, and it is no longer considered a rare or orphan disease (6). The prevalence of non-CF bronchiectasis in the general population has been estimated at 39.9 cases per 100,000 (7). In a study conducted at the largest tertiary hospitals in Ethiopia, non-CF bronchiectasis accounted for 5% of patients on follow-up at chest clinics, with a female predominance (8).

In the current management paradigm, one of the primary goals of bronchiectasis treatment is to reduce disease progression by interrupting the vicious cycle of exacerbation. Higher numbers of exacerbations are associated with accelerated decline in lung function and an increased risk of mortality. Pulmonary exacerbations are also linked to increased emergency department visits and hospitalization rates, leading to higher healthcare costs and substantial economic burden. Patients experiencing three or more exacerbations per year have poorer health status, are more likely to require hospitalization for treatment, and have a twofold higher mortality rate compared to those without exacerbations (9,16).

The European Respiratory Society guidelines for bronchiectasis identify exacerbation prevention as a key treatment objective and recommend long-term macrolide therapy and inhaled antibiotics for adults with frequent (three or more) exacerbations per year (10,12).

Given the significant impact of exacerbations, increasing attention has been directed toward identifying and correcting modifiable risk factors. A study conducted in Ukraine found that overweight, airway obstruction, longer disease duration, more severe dyspnea, greater number of affected pulmonary lobes, and the presence of one or more comorbidities were independently associated with frequent exacerbations (17).

In a developing country such as Ethiopia, where healthcare resources to address the consequences of frequent exacerbations are limited, identifying risk factors for frequent exacerbation is particularly important. To date, no comparative studies have been conducted in sub-Saharan Africa to identify independent risk factors associated with the frequent exacerbator phenotype.

This study therefore aims to evaluate factors affecting exacerbation frequency among non-CF bronchiectasis patients receiving follow-up care at Tikur Anbessa Specialized Hospital.

## Methods and Materials

Study Area and Study Population: A hospital-based comparative study was conducted among patients attending the Chest Clinic at Tikur Anbessa Specialized Hospital, the largest tertiary hospital in Ethiopia, between June 2023 and July 2024.

**Inclusion and exclusion criteria**: Inclusion criteria were patients aged ≥18 years with a diagnosis of non-CF bronchiectasis confirmed by high-resolution computed tomography.

Exclusion criteria included patients with cystic fibrosis bronchiectasis and those with active tuberculosis.

**Sample techniques and procedures**: A formal sample size calculation was not performed due to the limited number of bronchiectasis patients attending the study center. Given the small pool of eligible patients and the lack of similar prior local studies to inform assumptions, statistical power estimation was not feasible. Consequently, all eligible patients presenting during the study period were consecutively enrolled.

**Figure 1:**
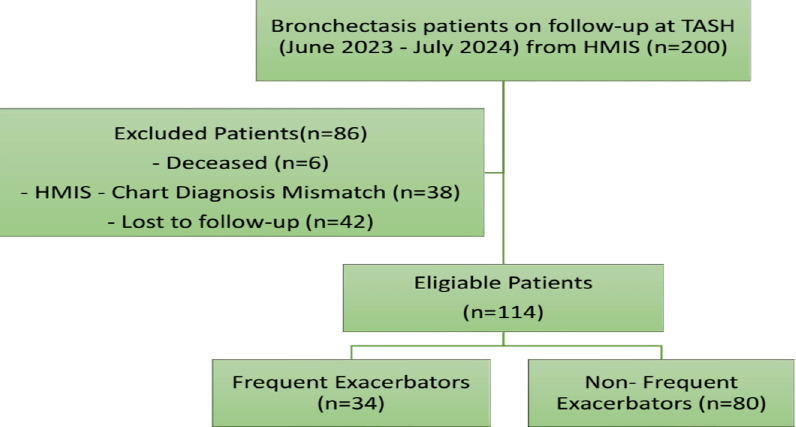
Patient eligibility flow diagram

Eligible participants were categorized into two groups in a ratio of 2.4:1 (Fig. 1):

⚬ **Group 1:** Non-frequent exacerbators, defined as <3 exacerbations per year.
⚬ **Group 2:** Frequent exacerbators, defined as ≥3 exacerbations per year.

**The following Operational definitions are used:**

⚬ **Non-cystic fibrosis bronchiectasis**: bronchiectasis not caused by cystic fibrosis.
⚬ **Bronchiectasis exacerbation**: deterioration in three or more of the following symptoms for at least 48 hours—cough; sputum volume and/or consistency; sputum purulence; breathlessness and/or reduced exercise tolerance; fatigue and/or malaise; hemoptysis—requiring a clinician-determined change in treatment.
⚬ **Case/Frequent exacerbators**: ≥3 exacerbations per year.
⚬ **Control/Non-frequent exacerbators**: <3 exacerbations per year.
⚬ **Demographic characteristics**: age, sex, and educational status.
⚬ **Socio-economic status**: social standing based on education, income, and occupation.
⚬ **Smoking history**: calculated as pack-years (packs per day × years smoked).
⚬ **Use of home oxygen**: intermittent or continuous oxygen use at home.
⚬ **Duration of illness**: time from initial bronchiectasis diagnosis to the current period.
⚬ **Cause of bronchiectasis**: presumed underlying etiology.
⚬ **Comorbidity**: presence of a distinct medical diagnosis alongside bronchiectasis.
⚬ **QOL-B**: patient-reported outcome measure assessing symptoms, functioning, and health-related quality of life in non-CF bronchiectasis.
⚬ **Previous hospitalization**: prior admission due to bronchiectasis exacerbation.
⚬ **Type of antibiotics during hospitalization**: antibiotics used during admission for exacerbation.
⚬ **Duration of hospitalization**: length of inpatient stay.
⚬ **Airway obstruction**: airflow limitation determined by spirometry.
⚬ **Long-term macrolide therapy**: use of a macrolide antibiotic for a duration of ⩾3 months.
⚬ **Bacterial colonization**: persistent presence and growth of bacteria in the airway.

**Data collection procedure**: A structured data collection tool was developed. Data were obtained from electronic medical records and direct patient communication via phone calls. Three trained data collectors participated in data collection after receiving formal orientation from the principal investigator. Data were collected using Kobo Toolbox.

**Data quality assurance**: The data collection tool was pretested on two participants to identify inconsistencies and errors; these participants were excluded from the final analysis. Necessary corrections were made prior to actual data collection.

**Statistical analysis**: Statistical analyses were conducted using SPSS version 26. Descriptive statistics summarised baseline characteristics. Associations between categorical variables and frequent exacerbations were assessed using Chi-square tests. Bivariate logistic regression was used to identify candidate predictors, with crude odds ratios (COR) calculated. Variables with p < 0.2 were included in the multivariable logistic regression model to control for confounding. Results were expressed as adjusted odds ratios (AOR) with 95% confidence intervals (CI). Variables with p < 0.05 were considered independent predictors of frequent exacerbations.

**Ethical consideration**: Ethical approval was obtained from the Institutional Review Board of Addis Ababa University, College of Health Sciences, Department of Internal Medicine (August 13, 2024; approval number not issued). Verbal informed consent was obtained from all participants after explaining the study objectives, procedures, and participant rights. Data were collected anonymously, and confidentiality was strictly maintained through secure, password-protected storage accessible only to the research team.

## Results

Among the 114 participants included in the study, 30% were frequent exacerbators, while 70% were non-frequent exacerbators (Fig. 1).

This figure illustrates the flow of patient screening and enrolment. Of the total patients evaluated at the Chest Clinic, 114 met the eligibility criteria and were grouped into two categories: frequent (exacerbations≥3/year) and non-frequent (exacerbations <3/year).

The mean age (±SD) was 49.54 ± 15.08 years. Females comprised 63 (55%) of participants, and 75 (65.8%) resided in Addis Ababa. Thirty-three percent had completed primary education, and 41.2% reported no monthly income. Twenty-three participants (20%) had a history of smoking, and 12 (10.5%) were obese. Forty participants (35%) had lived with bronchiectasis for more than three years, and post-tuberculosis bronchiectasis was the most common etiology, accounting for 75 cases (65.8%). Twenty-six participants (23%) had resting oxygen saturation <88%. Comorbid conditions were present in 85 participants (75%), with asthma being the most common (22, 25.9%), followed by hypertension (20, 23.5%), pulmonary hypertension (20, 23.5%), and HIV infection (12, 14.1%) (Table 1).

**Table 1:** Baseline characteristics of study participants with non-CF bronchiectasis

Variable	N(%)	Bronchiectasis Frequent Exacerbation		Chi-square (p-value)

		Yes	No	
Age in years				
18-35	23(20.2)	6(26.1)	17(73.9)	12.9(0.005)
36-50	33(28.9)	6(18.2)	27(81.8)	
51-65	32(28.1)	7(21.9)	25(78.1)	
>65	26(22.8)	15(57.7)	11(42.3)	
Gender				
Female	63(55.3)	17(27)	46(73)	0.54(0.461)
male	51(44.7)	17(33.3)	34(66.7)	
Address				
Addis Ababa	75(65.8)	14(18.7)	61(81.3)	13.04(<0.001)
Out of Addis Ababa	39(34.2)	20(51.3)	19(48.7)	
Educational status				
No formal education	24(21.1)	16(66.7)	8(33.3)	
Primary	38(33.3)	5(13.2)	33(86.8)	21.29(<0.001)
Secondary	34(29.8)	9(26.5)	25(73.5)	
Collage and above	18(15.8)	4(22.2)	14(77.8)	
Smoking history				
Yes	23(20.2)	13(56.5)	10(43.5)	9.81(0.002)
No	91(79.8)	21(23.1)	70(76.9)	
Duration bronchiectasis in years				
≤1	38(33.3)	5(13.2)	33(86.8)	12.86(<0.001)
1-3	36(31.6)	8(22.2)	28(77.8)	
>3	40(35.1)	21(52.5)	19(47.5)	
Etiology of bronchiectasis				
Congenital	3(2.6)	3	0	14.39(0.1099)
Fibrosis	6(5.3)	2(33.3)	4(66.7)	
Post Tuberculosis	75(65.8)	22(29.3)	53(70.7)	
Post infection	5(4.4)	1(20)	4(80)	
Others	25(21.9)	6(24)	19(76)	
Resting saturation				
<88	26(22.8)	15(57.7)	11(42.3)	12.49(0.000)
≥88	88(77.2)	19(21.6)	69(78.4)	
Types of comorbid disease (n=85)				
Hypertension	20(23.5)	6(30)	14(70)	1.24(0.947)
Diabetes Mellitus	7(8.2)	1(14.3)	6(85.7)	0.841(0.356)
Valvular heart disease	9(7.9)	3(33.3)	6(66.7)	0.872(0.785)
Pulmonary hypertension	20(23.5)	5(25)	15(75)	0.245(0.620)
HIV	12(14.1)	4(33.3)	8(66.7)	0.104(0.748)
malignancy	8(9.4)	3(37.5)	5(62.5)	1.28(0.598)
asthma	22(25.9)	12(54.5)	10(45.5)	9.03(0.003)
Chronic Obstructive Pulmonary Disease	7(8.2)	2(28.6)	5(71.4)	5.93 (0.015)
Others	39(45.9)	4(10.3)	35(89.7)	

Ninety-six participants (84%) were receiving medications, with mucolytics being the most commonly prescribed (68, 70.8%), followed by bronchodilators and inhaled corticosteroids. Seventeen participants (15%) were on long-term macrolide therapy. Fourteen participants (14.6%) were receiving long-term home oxygen therapy, representing nearly 50% of those with resting saturation <88% (Table 2).

**Table 2:** Medication history related characteristics of the study participants

	n(%)	Bronchiectasis Frequent Exacerbation		Chi-square (p-value)
Variable		Yes	No	
Medication history				
Yes	96(84.2)	32(33.3)	64(66.7)	3.58(0.059)
No	18(15.8)	2(11.1)	16(88.9)	
The types of medication used(n=96)				
Mucolytics	68(70.8)	27(39.7)	41(60.3)	4.26(0.039)
Bronchodilators	50(52.1)	18(36)	32(64)	1.33(0.563)
Inhaled corticosteroids	40(41.7)	16(40)	24(60)	1.37(0.242)
Long-term macrolide therapy	17(17.7)	9(52.9)	8(47.1)	3.57(0.059)
Home oxygen	14(14.6)	9(64)	5(36)	2.05(0.152)
Use of Long-term macrolide therapy				
Yes	17(14.9)	9(52.9)	8(47.1)	5.10(0.024)
No	97(85.1)	25(25.8)	72(74.2)	
Types of Long-term macrolide therapy(n=17)				
Azithromycin	17(100)	9(52.9)	8(47.1)	6.23(0.044)
Duration of Long-term macrolide therapy				
3-6month	1(5.9)	0	1	6.70(0.082)
6-12month	6(35.3)	3(50)	3(50)	
>12month	10(58.8)	6(60)	4(40)	

Sputum culture was performed for 86 participants (75.4%). No bacterial growth was observed in 60.5%, while Pseudomonas aeruginosa was isolated in 10 participants (11.6%). Eight participants with P. aeruginosa received eradication therapy, with cefepime monotherapy being the most commonly used regimen (Table 3).

**Table 3:** Microbiologic assessment related characteristics of the participants

Variable	N (%)	Bronchiectasis Frequent Exacerbation		Chi-square(p-value)

		Yes	No	
Sputum culture done				
Yes	86(75.4)	26(30.2)	60(69.8)	0.867(0.028)
no	28(24.6%)	8(28.6)	20(71.4)	
Sputum culture results(n=86)				
Actinobacteria specious	1(1.2)	1	0	15.3(0.053)
candida	1(1.2)	0	1	
E. coli	6(6.9)	3(50)	3(50)	
Klebsiella pneumoniae	3(3.5)	0	3(100)	
No growth	52(60.5)	11(21.2)	41(78.8)	
others	12(12.9)	4(33.3)	8(66.7)	
Pseudomonas aeruginosa	10(11.6)	7(70)	3(30)	
Staphylococcus species	1(1.2)	0	1	
History of Pseudomonas eradication therapy (n=10)				
Yes	8(80)	5(62.5)	3(37.5)	6.53(0.009)
no	2(20)	2	0	
Antibiotic used for eradication (n=8)				
Cefepime	4			
Ciprofloxacin	1			
Meropenem	1			
Cefepime + Ciprofloxacin	2			

The mean St. George's Respiratory Questionnaire (SGRQ) score among frequent exacerbators was 21.2, compared with 25.30 among non-frequent exacerbators (Table 4). Based on the Medical Research Council (MRC) Dyspnea Scale, 44 participants (38.6%) were grade I, while 18 (16%) had grades III or IV dyspnea (Table 5).

**Table 4:** Quality of life scores among bronchiectasis patients based on St. George's Respiratory Questionnaire

Quality of life total score						p-value
Frequent exacerbators of bronchiectasis	Mean	N	Std. Deviation	Minimum	Maximum	
No	25.30	80	4.535	11	30	<0.001
Yes	21.26	34	6.321	8	30	
Total	24.10	114	5.430	8	30	
	

Lower SGRQ scores indicate worse health-related quality of life.

**Table 5:** Functional status of study participants based on dyspnea grading and pulmonary function tests

Variable	N (%)	Bronchiectasis Frequent Exacerbation		Chi square (p-value)

		yes	No	
Dyspnea Grading (Based on MRCDS)				
Grade 0	36(31.6)	4(11.1)	32(88.9)	16.1(0.003)
Grade I	44(38.6)	12(27.3)	32(72.7)	
Grade II	16(14)	7(43.8)	9(56.3)	
Grade III	10(8.8)	6(60)	4(40)	
Grade IV	8(7)	5(62.5)	3(37.5)	
Pulmonary function test done				
Yes	54(47.4)	15(27.8)	39(72.2)	0.21(0.650)
no	60(52.6)	19(31.7)	41(68.3)	
FEV1& FVC ratio				
<0.7	23	6(27.3)	16(72.7)	0.129(0.938)
≥0.7	31	9(29)	22(71)	
Airflow limitation stages in patients with FEV1/FVC ratio < 0.7				
Stage 1 (FEV1>=80% predicted)	1(4.5)	0	1	1.34(0.854)
Stage 2 (FEV1 50%-80% predicted)	9(40.9)	3(33.3)	6(66.7)	
Stage 3 (FEV1 30%-50% predicted)	10(45.5)	2(20)	8(80)	
Stage 4 (FEV1 <30% predicted)	2(9.1)	1(50)	1(50)	

FEV1: Forced Expiratory Volume in 1 Second; FVC: Forced Vital Capacity

In bivariate logistic regression analysis, age, residence, educational status, smoking history, resting oxygen saturation, asthma, and dyspnea grade were associated with frequent exacerbations at a significance level of p < 0.2. In multivariable analysis, age, residence, educational status, smoking history, resting oxygen saturation, asthma, and dyspnea grade remained independent determinants of the frequent exacerbator phenotype. Participants aged >65 years had 3.7-fold higher odds of frequent exacerbations compared to those aged 18–35 years (AOR = 3.7, 95% CI: 2.87–19.47). Those residing outside Addis Ababa had 9.7-fold higher odds compared to urban residents (AOR = 9.7, 95% CI: 2.35–40.07). Participants without formal education had 17.2-fold higher odds of frequent exacerbations compared to those with college-level education or higher (AOR = 17.2, 95% CI: 1.78–66.98). A history of smoking increased the odds of frequent exacerbation by 6.2 times (AOR = 6.2, 95% CI: 1.25–31.15). Resting oxygen saturation <88% was associated with a 10.1-fold increased risk (AOR = 10.1, 95% CI: 1.87–54.73). Comorbid asthma increased the odds by 4.3 times (AOR = 4.3, 95% CI: 1.34–16.41). Participants with dyspnea grades III and IV had 8.6- and 14.2-fold higher odds, respectively, compared to those with grade 0 dyspnea (Table 6).

**Table 6:** The bivariate and multivariate association between independent variable and Bronchiectasis frequent exacerbation

variable	Bronchiectasis Frequent Exacerbation		p-value	COR with 95%CI	P-value	AOR with 95%CI

	Yes	No				
Age in years						
18-35	6(26.1)	17(73.9)	1		1	
36-50	6(18.2)	27(81.8)	0.480	0.63(0.17, 2.27)	0.621	0.60(0.08, 4.45)
51-65	7(21.9)	25(78.1)	0.717	0.79(0.23, 2.78)	0.834	1.2(0.18, 8.62)
>65	15(57.7)	11(42.3)	0.029	3.9(1.15, 12.99)	0.003	3.7(2.89, 19.47)
Address						
Addis Ababa	14(18.7)	61(81.3)	1		1	
Out of Addis Ababa	20(51.3)	19(48.7)	0.000	4.6(1.95, 10.79)	0.002	9.7(2.35, 40.07)
Educational status						
No formal education	16(66.7)	8(33.3)	0.006	7(1.73, 28.34)	0.014	17.2(1.78, 66.98)
Primary	5(13.2)	33(86.8)	0.393	0.53(0.12, 2.27)	0.389	0.35(0.03, 3.87)
Secondary	9(26.5)	25(73.5)	0.737	1.3(0.33, 4.85)	0.131	5.4(0.60, 49.20)
Collage and above	4(22.2)	14(77.8)	1		1	
Smoking history						
Yes	13(56.5)	10(43.5)	0.003	4.3(1.66, 11.29)	0.006	6.2(1.25, 31.15)
No	21(23.1)	70(76.9)	1		1	
Resting saturation						
<88	15(57.7)	11(42.3)	0.001	4.9(1.96, 12.54)	0.007	10.1(1.87, 54.73)
≥88	19(21.6)	69 (78.4)	1		1	
Asthma						
No	13(20.6)	50(79.4)	1		1	
Yes	12(54.5)	10(45.5)	0.004	4.6(1.64, 13.03)	0.012	4.3(1.34, 16.41)
Dyspnea Grading						
Grade 0	4(11.1)	32(88.9)	1		1	
Grade I	12(27.3)	32(72.7)	0.081	3.0(0.87, 10.29)	0.124	2.9(0.59, 11.24)
Grade II	7(43.8)	9(56.3)	0.012	6.2(1.48, 26.10)	0.244	5.6(0.14, 12.65)
Grade III	6(60)	4(40)	0.003	12(2.33,61.70)	0.014	8.6(4.24, 48.44)
Grade IV	5(62.5)	3(37.5)	0.004	13.3(2.27, 78.19)	0.021	14.2(3.47, 62.42)

## Discussion

In this study, 114 patients were recruited, of whom 30% were classified as frequent exacerbators, experiencing three or more exacerbations per year. This finding is comparable to large studies from Europe and Australia, which reported proportions of frequent exacerbators of 37% and 23%, respectively (18,19). These results highlight the substantial burden of the frequent exacerbator phenotype, which is associated with poorer clinical outcomes and reduced quality of life, underscoring the need for effective risk stratification and early intervention in high-risk groups.

Age greater than 65 years was identified as an independent predictor of frequent exacerbations (AOR = 3.7). This is consistent with previous studies demonstrating that ageing is associated with dysfunction of both innate and adaptive immunity, leading to increased susceptibility to chronic infections and reduced respiratory reserve. Additionally, age-related declines in mucociliary clearance and the accumulation of comorbidities contribute to recurrent infections and exacerbations among older adults. The elevated risk observed in this population underscores the importance of proactive monitoring, including vaccination, pulmonary rehabilitation, and consideration of long-term macrolide therapy where appropriate (20,21).

Residence outside Addis Ababa was also strongly associated with frequent exacerbations (AOR = 9.7). This association likely reflects challenges common in tuberculosis-endemic, resource-limited settings, including limited access to specialized healthcare, delays in diagnosis, under-treatment, and increased exposure to environmental irritants such as biomass smoke in rural areas. For instance, a systematic review of post-tuberculosis lung disease in sub-Saharan Africa reported higher symptom prevalence in rural settings (68.8%) compared with urban areas (39.1%) (22). Furthermore, studies from rural Uganda have documented substantial barriers to diagnostic tools, guideline-based care, and specialist training relative to urban centers (23). These rural-urban disparities remain major obstacles to the optimal management of chronic lung diseases in low- and middle-income countries (24). Addressing these challenges requires strengthening healthcare delivery in rural and remote areas through guideline adaptation, clear referral pathways, community-based education, and improved access to specialist care.

Lack of formal education emerged as the strongest independent predictor of frequent exacerbations (AOR = 17.2). Health literacy plays a critical role in disease management, particularly in bronchiectasis, where treatment often involves complex medication regimens, airway clearance techniques, and lifestyle modifications. Limited literacy may impede patients' understanding of treatment plans, leading to poor adherence and worsening disease control. This is supported by evidence showing that individuals with poor reading ability face significant difficulties accessing healthcare, understanding recommended treatments, and following provider instructions (25). Given the importance of both pharmacological and non-pharmacological interventions in reducing exacerbations, providing clear, concise, and age-appropriate educational materials—using written instructions, visual aids, and multimedia resources—may help address literacy-related barriers.

A history of smoking was significantly associated with frequent exacerbations (AOR = 6.2). A cohort study has shown that individuals with a history of smoking and suspected bronchiectasis, even with normal spirometry, have a 15% increased risk of all-cause mortality (27). Smoking cessation is therefore a cornerstone of bronchiectasis management due to its detrimental effects on disease progression and associated comorbidities. Targeted cessation programs, incorporating technology-based interventions and focused education for high-risk groups, are essential to mitigate the impact of smoking (26).

Resting hypoxemia, defined as oxygen saturation below 88%, was a strong independent predictor of frequent exacerbations (AOR = 10.1), reflecting more severe underlying disease. Hypoxemia compromises host defense mechanisms, increasing susceptibility to infections and other triggers of exacerbations. Previous studies have demonstrated that lower resting oxygen saturation is associated with a higher risk of severe exacerbations, and exercise-induced desaturation during tests such as the six-minute walk test has similarly been linked to disease severity and exacerbation risk (28,29). Patients with hypoxemia may benefit from long-term oxygen therapy, closer clinical monitoring, and preventive antimicrobial strategies. These findings also highlight the need for improved availability of pulse oximetry in hospitals across Ethiopia, where access remains limited.

Asthma was another significant contributor to frequent exacerbations (AOR = 4.3). Asthma is a common comorbidity in patients with bronchiectasis and has been consistently shown to increase the risk of exacerbations (30,31). The asthma–bronchiectasis overlap phenotype, well described in the literature, presents particular management challenges (32). Patients with coexisting asthma and bronchiectasis require a tailored approach, including optimized asthma diagnosis and management, supported by wider availability of spirometry and appropriate pharmacotherapy.

Patients with dyspnea grades III and IV had substantially higher odds of frequent exacerbations (AOR = 8.6 and 14.2, respectively). Dyspnea serves not only as a symptom but also as an indicator of disease severity and functional impairment. Studies have shown that patients with more severe dyspnea experience more frequent exacerbations, require greater use of respiratory medications, and have worse lung function (33). Severity indices such as the Bronchiectasis Severity Index further validate dyspnea as a strong predictor of exacerbation frequency, hospitalization, and mortality (34). Management of patients with high dyspnea grades should therefore be multidimensional, incorporating long-acting bronchodilators, nutritional support, airway clearance techniques, and long-term oxygen therapy for those with hypoxemia.

Pseudomonas aeruginosa colonization was more common among frequent exacerbators (p = 0.006), consistent with previous studies demonstrating its role in increasing exacerbation risk and supporting the use of eradication therapy (35,36). However, it did not remain an independent predictor in multivariable analysis, likely due to the limited sample size.

This study has several limitations. The relatively small sample size and absence of a formal sample size calculation may have reduced statistical power and limited generalizability, particularly for variables such as Pseudomonas colonization. As a comparative cross-sectional study, the findings demonstrate associations rather than causality, although they provide valuable insights that warrant confirmation in prospective longitudinal studies. Data collection through phone interviews and medical record review may have introduced recall or documentation bias, particularly regarding exacerbation history and smoking exposure. Wide confidence intervals for some predictors reflect imprecision due to small subgroup sizes. Additionally, because frequent exacerbators constituted only 30% of the cohort, the multivariable model may be susceptible to overfitting and should be interpreted with caution.

Despite these limitations, the findings have important clinical and public health implications in Ethiopia. Identifying frequent exacerbators enables more efficient allocation of limited resources, including pulmonary rehabilitation, vaccination, and Long-term macrolide therapy. Integrating bronchiectasis care into existing post-tuberculosis and chronic lung disease programs may enhance continuity of care and optimize the use of scarce diagnostic and therapeutic resources. Local adaptation of clinical guidelines, with emphasis on early risk assessment, smoking cessation, and community-level education, is essential.

In summary, nearly one-third of patients with bronchiectasis receiving follow-up at TASH experience frequent exacerbations. These are driven by both modifiable factors—such as smoking, hypoxemia, and uncontrolled asthma—and non-modifiable factors, including older age, rural residence, and low educational attainment. Identification of these determinants allows for targeted interventions: modifiable risks can be addressed through clinical management, while non-modifiable factors highlight vulnerable populations who require prioritized monitoring and education-based support. Implementing simple risk stratification strategies at the clinic level and integrating bronchiectasis care into post-tuberculosis and chronic lung disease programs may reduce exacerbation burden, optimize resource use, and improve outcomes in resource-limited settings.
